# What makes a good surgical experience for the naïve learner?

**DOI:** 10.15694/mep.2018.0000097.1

**Published:** 2018-05-09

**Authors:** Daniel Axelrod, Eric Walser, Graeme Hoit, Jason Y. Lee

**Affiliations:** 1 McMaster University; 2 University of Western Ontario; 3 University of Toronto; 4 University Health Network

**Keywords:** Observership, Surgical Education, Career Exploration, Mentor, Shadowing

## Abstract

This article was migrated. The article was marked as recommended.

Early clinical observerships play a key role in pre-clerkship education and career selection. Using a cross sectional survey design, we attempted to assess the makeup of the student’s observership throughout their time in the operating room (OR). Perceived educational value (EV), utility in career exploration (CE), and level of personal enjoyment (PE) were assessed after every encounter and utilized as primary outcomes.

Twenty-eight (28) 1st year medical students participating in an intensive 2-week surgical exploration program completed eight 34 question electronic surveys characterizing each of their 8 surgical observerships (224 events). One hundred forty six (65.2%) surveys were completed, each representing a day of observerships, with a total of 207 surgeries observed.

Following multivariate linear regression analysis, increased surgical team engagement with the student and a positive tone of interaction were each significantly associated with improved EV (p1 = 0.013, p2 <0.001), CE (p1=0.006, p2=0.012), and PE (p1 <0.001, p2 <0.001). Surgical subspecialty, type of case and ability to scrub in were not associated with improved experiences.

Increased engagement and positive interaction with the surgical team are significantly associated with various measures of improved surgical experience, and each are highly modifiable factors in a learner’s OR experience. This research emphasizes the diverse educational responsibility of academic surgeons.

## Introduction

Choosing a specialty is often a difficult and frustrating process for medical students. For some, a decision is made early in their pre-clinical years[i] ^,[ii]^. However, for most this decision is a fluid process that can continue to shift until their final clinical year[iii] ^,[iv],[v],[vi]^. For all, their decision represents a collection of ideas, beliefs and values about medical specialties shaped by their pre-clerkship education, mentorship and clinical experiences throughout medical school[vii] ^,[viii]^.

A variety of factors, including concern over future employment opportunities and preference for controllable lifestyles specialties, have contributed to a decline in total applications to surgical programs across North America[ix] ^,[x]^. It has been demonstrated that many students who do apply for surgical residencies had positive medical school experiences in surgery, either through mentorship, observerships and research[xi] ^,[xii]^.

In 2012, the Surgical Exploration and Discovery program (SEAD) program was created by the University of Toronto Department of Surgery with an aim to provide pre-clerkship students a richer and earlier exposure to a variety of direct entry surgical specialties. The program provides a well- controlled setting for the naïve learner to explore the operating room (OR) and participate in the surgical experience[xiii].

The two-week SEAD program, which runs in the summer between first and second year of medical school, includes three main components: observerships, didactic presentations and technical skills workshop. Each of the 28 students involved completed 8 OR observerships in each surgical discipline at an academic or community hospital. The didactic presentations were either faculty or resident led sessions for each surgical specialty, taking place over lunch. Content focus was non- technical information including training path, scope of practice, daily responsibilities and research. Lastly, each afternoon a workshop was held in an accredited surgical skills lab focusing on technical skills and case management.

The purpose of this study was to determine what factors, either pre-operative, intraoperative, or post-operative, affected the operating room experience for naïve learners.

[i] Ellsbury, Kathleen E., and Frank T. Stritter. “A study of medical students’ specialty-choice pathways: trying on possible selves.” *Acad Med* 72 (1997): 534-541.

[ii] Erzurum VZ, Obermeyer RJ, Fecher A, et al. What influences medical students’ choice of surgical careers. Surgery. 2000;128(2):253-256.

[iii] Scott IM, Matejcek AN, Gowans MC, Wright BJ, Brenneis FR. Choosing a career in surgery: factors that influence Canadian medical students’ interest in pursuing a surgical career. Can J Surg. 2008;51(5):371-377

[iv] O’Herrin JK, Lewis BJ, Rikkers LF, Chen H. Why do students choose careers in surgery? J Surg Res. 2004;119(2):124-129

[v] Minor S, Poenaru, D *et al.* A study of career choice patterns among Canadian medical students. *Am J Surg. 2003 Aug; 186(2):182-8.*

[vi] Bland, Kirby I., and George Isaacs. “Contemporary trends in student selection of medical specialties: the potential impact on general surgery.” *Archives of Surgery* 137.3 (2002): 259-267.

[vii] Jagsi, Reshma, et al. “Sex, role models, and specialty choices among graduates of US medical schools in 2006-2008.” *Journal of the American College of Surgeons* 218.3 (2014): 345-352.

[viii] Kozar RA, Lucci A, Miller CC, et al. Brief intervention by surgeons can influence students toward a career in surgery. J Surg Res. 2003;111(1):166-169.

[ix] R-1 Match Reports - 2016. In: *CaRMS* [website of the Canadian Residency Matching Service]. Available: http://www.carms.ca/en/data-and-reports/r-1/reports-2016/ (accessed 2016 Jul 15).

[x] Marschall, JG *et al.* Decline in popularity of general surgery as a career choice in North America: review of postgraduate residency training selection in Canada, 1996-2001. *AAWorld J Surg. 2003 Mar; 27(3):249-52.*

[xi] O’Herrin JK, Lewis BJ, Rikkers LF, Chen H. Medical student operative experience correlates with a match to a categorical surgical program. Am J Surg. 2003;186(2): 125.

[xii] Cochran A, Paukert JL, Neumayer LA. Does a general surgery clerkship influence student perceptions of surgeons and surgical careers? Surgery. 2003;134(2): 153-157.

[xiii] Gawad, Nada, et al. “Planting the ‘SEAD’: early comprehensive exposure to surgery for medical students.” *Journal of surgical education* 70.4 (2013): 487-494.

## Materials and Methods

### Participants and Study Design

The study was designed as an optional program to run along SEAD, and participation did not affect the SEAD experience for students. Institutional ethics approval was received from the University of Toronto, Office of Research Ethics under protocol reference #27653. The study was explained to the participants by the investigators during the SEAD orientation session and accompanying written information was provided. Informed consent was obtained and documented.

Surgical faculty, residents, senior medical students involved in surgical education, and prior SEAD participants generated study surveys using a systematic approach of item generation and reduction (Appendix A).

### Testing

Prior to the first observerships, participants were asked to complete a baseline questionnaire detailing their demographic information, interest in a surgical career and prior experience in surgery. These characteristics were later incorporated into the statistical analysis.

Immediately following completion of the observership, students received links to complete electronic surveys detailing their experience via email. Each survey contained 34 questions, detailing cases of the day, involvement in said cases, preparation for the day and interaction with the surgical team. Students were asked to assess the experience in terms of surgical educational value (EV), career exploration value (CE) and their personal enjoyment of their experience (PE). Links remained active for 3 business days following distribution. These three self- reported outcomes (EV, CE, PE) represented the three primary endpoints of the study, and all modifiable variables were compared against them.

### Statistical Analysis

Descriptive statistics were performed using Microsoft Excel 2011. Statistical analysis was performed using SPSS **^®^** (IBM, Chicago, Illinois. 2002). Continuous and discrete data were compared with student t tests for independent groups. Multiple linear regressions with a general linear model was used to test for interactions between demographic, preoperative, intraoperative and postoperative variables with the three primary outcomes listed above (EV, CE, PE). P values that were less than 0.05 were regarded as significant.

## Results

We recruited 28 participants and distributed 224 surveys, of which 146 surveys were completed in full and analyzed (response rate 65.1%). Demographic variables are listed in Table 1. Of note, 85.8% of participants were younger than 25, 68% were male, and a near majority of participants (49.9%) had previously observed more than 6 surgeries during their medical training.

**Table 1.** Participants Characteristics (n=28)

	Group	Total
Age		
	20-22	7 (25.0%)
	23-25	17 (60.1%)
	>25	4 (14.2%)
Gender		
	Male	19 (67.8%)
	Female	9 (32.1 %)
Level of education		
	Bachelors	24 (85.7%)
	Masters	4 (14.2%)
Number of surgeries previously observed		
	0	1 (3.5%)
	1 to 5	13 (46.4%)
	6 to 10	4 (14.2%)
	11 to 20	7 (25.0%)
	21+	3 (10.7%)
Number of cases previously scrubbed in		
	0	9 (32.1%)
	1 to 5	12 (42.8%
	6 to 10	3 (10.7%)
	11+	4 (14.2%)
Previously participated in surgical workshops		
	Yes	12 (44.4%)
	No	15 (55.6%)
Interest in surgery as a career		
	Very	13 (46.4%)
	Somewhat	14 (50.0%)
	Low	1 (3.6%)
	Not	0

Regarding the observership experience ( Table 2), General surgery (16.1% of all observerships), Neurosurgery (13.1%), Orthopaedics (13.1%) and Urology (13.1%) represented the best surveyed operating rooms. The large majority of operations were held in an academic hospital setting (n=135, 94.4%), with more than 2 other learners present (n=181, 89.4%), and either minimally invasive or mixed open/laparoscopic cases (n= 154, 74.4%). Most students reported preparing for their case (n=88, 60.7%) while students were able to scrub into the case approximately every third operation (n=73, 35.4%). Conversely, 33 students (22.6%) served as either the 1 ^st^ or 2 ^nd^ assist during an operation.

**Table 2.** Characteristics of Observerships

		Count	Percentage
Specialty observed		Count (n)	% of total
n = 143	Cardiac Surgery	13	9.0
	General Surgery	24	16.6
	Neurosurgery	19	13.1
	Orthopaedic Surgery	19	13.1
	Plastic Surgery	14	9.7
	Urologic Surgery	19	13.1
	Vascular Surgery	14	9.7
	Thoracic Surgery	12	8.3
	Paediatric Surgery	11	7.6
Hospital type			
n=143	Academic	135	94.4
	Community	8	5.6
Other learners present			
n=203	1	20	9.8
	2	88	43.5
	3+	95	46.8
Case Type			
n=207	Open	46	22.2
	Minimally Invasive	131	63.3
	Mixed	7	3.4
	Converted	23	11.1
Did you prepare ahead of time?			
N =145	Yes	88	60.7
	No	57	39.3
Observer scrubbed in?			
n =206	Yes	73	35.4
	No	133	64.6

The students reported mean educational value (EV) of their experience as 3.64/5 (SD = +/-1.04), value in career exploration (CE) as 3.76/5 (SD = +/-0.93) and personal enjoyment (PE) as 3.82/5 (SD = +/- 1.00). Following multivariate analysis ( Table 3) and linear regression, two independent variables were reported as being significantly associated with increased EV, CE and PE ( Table 3A- C). These were 1) increased surgical team engagement with the student (EV, p=0.013. CE, p=0.006. PE, p = <0.001) and 2) positive tone of interaction while in the operating room (EV, p=<0.001), CE, p =0.012. PE, p < 0.001).

Additionally, the student’s involvement with the case was independently significantly associated with personal enjoyment (p=0.03), while trending towards significance in terms of increased educational value (p=0.143) and utility in career exploration (p= 0.058).

Of note, surgical subspecialty, prior knowledge of cases or preparation, type of case and ability to scrub in were not associated with improved experiences in any of three primary outcomes evaluated.

**Table 3A.** Utility for Career Exploration

Variable	F-test	p-value
Scrubbing In	0.925	0.435
No. of team members	0.483	0.696
Length of case	1.302	0.284
Prior preparation	0.23	0.875
Involvement in case	2.658	0.058
Team engagement	**4.663**	**0.006**
Positivity of interaction	**4.037**	**0.012**

**Table 3B.** Perceived Educational Value

Variable	F-test	p-value
Scrubbing In	1.321	0.277
No. of team members	1.061	0.374
Length of case	1.121	0.349
Prior preparation	1.067	0.371
Involvement in case	1.889	0.143
Team engagement	**3.432**	**0.023**
Positivity of interaction	**3.953**	**0.013**

**Table 3C.** Personal Enjoyment

Variable	F-test	p-value
Scrubbing In	0.801	0.499
No. of team members	0.498	0.685
Length of case	0.453	0.716
Prior preparation	1.461	0.236
Involvement in case	**3.203**	**0.03**
Team engagement	**11.134**	**>0.001**
Positivity of interaction	**11.032**	**>0.001**

## Discussion

A pre-clerkship observership is a common method for medical students interested in a career to explore a specific subspecialty[i]. With limited exposure to surgical specialties in pre-clerkship and little formal surgical teaching, the observership represents a rare potential opportunity to positively influence medical students towards a career in surgery before selecting their ultimate career path[ii] ^,[iii]^. The OR, however, is often a rigid and intimidating environment, which can be difficult to navigate for junior medical students. It was our goal to examine what aspects of an operating room experience yielded utility towards career exploration, personal enjoyment and educational value. There has been little research exploring this key moment in a potential surgeon’s early career.

Traditionally, the operative room has been seen as a challenging environment for junior students as it is orderly, structured and unfamiliar to those beginning medical school. It is often empirically assumed that if the student is able to be involved in the case, whether by scrubbing in or actually performing part of the surgical tasks (suturing, retracting, etc), that they would consider the experience successful and may be more likely to return to the OR or ultimately pursue a career in surgery ^10,[iv]^. Similarly, it has been assumed that the type of operation might influence a student’s experience. For example, a closed operation completed through laparoscopic techniques may afford the student a better view of the operating field, but less hands-on experience. Moreover, the number of learners in the operating room had often been seen as relevant to the junior medical students OR experience. Logically, with more learners, the most junior student may not be able be involved with more technical responsibilities during the case (holding retractors, suturing, use of cautery), or experience real or perceived barriers to asking questions.

Our research is the first to objectively characterize the above-mentioned assumptions. Interestingly, the junior medical students experience is neither significantly influenced by their ability to scrub into the case, the number of learners in the operating room, nor the type of case (open or closed). The only relevant factors which influence the student’s perceived educational value, utility in career exploration and personal enjoyment were 1) positive team engagement and 2) a positive tone in team interaction.

There is no clear or obvious explanation to our findings, but we hypothesize the outcomes are produced from reciprocated respect and interest between the student and surgical team. With a positive tone of interaction and significant team engagement with the student, the team is reciprocating the junior learner’s attention and interest in the case. The surgical team recognizes the interest the student has shown in their career, and chooses to assist them to maneuver through the challenging OR environment through engagement and positive tone. Additionally, the more the learner is engaged, the more respected he or she may feel, and the more likely they are to continue with their investment of time or attention towards the operation. Simply scrubbing into the case, or having only a few learners present does not guarantee that the learner will be given any attention or focus from the senior residents or attending surgeon.

In a similar vein, there was a near statistically significant association between involvement in the case and the three above mentioned outcomes. If a student has the ability to perform a technical task, it is likely the result of continued attention throughout the case, as well as mutual respect from the surgical team. The surgical team understands the time commitment the student has put towards this experience, and will offer technical tasks (such as cutting sutures, or suturing) to reciprocate their effort.

We believe these findings are extremely relevant to medical and surgical education practices. For a good observership experience, it need not be an absolute requirement that there are few students in the operating theatre, nor that the students need to be scrubbed in. Rather, a good surgical experience can be gained merely through mutual respect and attention from the attending team towards the medical student. If generalizable to all students, this intervention may be a cost neutral and resource efficient approach to raising interest and ultimately applicants towards surgical residencies that have seen a drop in interest[v].

It remains to be seen whether the importance of engagement and positive tone is preserved as trainees’ progress through the training hierarchy. It may be that as a learner progresses there technical skills take on more importance towards their ultimate enjoyment and educational value. This research is especially relevant to junior surgical residents who frequently are not the primary operator. This represents an important area for further study.

As in all cross-sectional studies, there are numerous limitations in our research. Firstly, we are prone to non-responder bias, though our response rate was satisfactory at 65.2%. Secondly, it is difficult to draw causation from results in a survey due to inability to find temporal relationships to independent variables. However, in our research the surveys were systematically administered following each exposure and thus our ability to find causation is higher than in most surveys. Moreover, though we had 146 surveys completed, our study population was quite small and limited by the participants in the SEAD program. This yielded an underpowered study which lead to difficulty finding statistical significance. Lastly, our population of respondents included only students who already had an existing interest in pursuing a surgical specialty, which cannot be extrapolated to all medical students. This limitation affects the external validity of the study, but can still serve as a reference for future research.

[i] Kost, Amanda, et al. “Primary care residency choice and participation in an extracurricular longitudinal medical school program to promote practice with medically underserved populations.” *Academic Medicine* 89.1 (2014): 162-168.

[ii] Glynn RW, Kerin MJ. Factors influencing medical students and junior doctors in choosing a career in surgery. Surgeon. 2010;8(4):187-191.

[iii] Azizzadeh A, McCollum CH, Miller CC III, Holliday KM, Shilstone HC, Lucci A Jr. Factors influencing career choice among medical students interested in surgery. Curr Surg. 2003;60(2):210-213.

[iv] Riboh J, Curet M, Krummel T. Innovative introduction to surgery in the preclinical years. Am J Surg. 2007;194(2):227-230.

[v] Pugno PA, McPherson DS, Schmittling GT, Kahn NB Jr. Results of the 2001 National Resident Matching Program: family practice. *Fam Med.* 2001;33:594-601.

## Conclusions

Frequent and positive team engagement significantly impacted career exploration, personal enjoyment and educational value during a surgical observership, while many other factors did not affect the student’s experience in this cohort. Though this study represents findings amongst a cohort of medical students with a previous interest in surgery, the results may be generalizable to all medical students with further study.

These findings emphasize that early in a student’s exposure to the OR, preceptor engagement is more important than involvement in the operation. Interventions aiming to increase intraoperative teaching by all team members represent low-cost, high-impact interventions that may yield to higher interest in surgical subspecialties and ultimately more medical student pursuing these professions.

## Take Home Messages

Frequent and positive team engagement influences career exploration, personal enjoyment and educational value of an observership.

Early in a student’s exposure to the OR, preceptor engagement is more important than involvement in the operation.

This may represent a novel direction to increase interest in surgical residency and create surgical mentorship.

## Notes On Contributors

Daniel Axelrod is an Orthopaedic resident at McMaster University, Hamilton, Ontario, Canada

Eric Walser is a General Surgery resident at University of Western Ontario, London, ON, Canada

Graeme Hoit is an Orthopaedic resident at the University of Toronto, ON, Canada

Jason Y. Lee is a staff urologist at the University Health Network, Toronto, ON, Canada
