# Supplementary material for: What makes a good surgical experience for the naïve learner?
Source: MedEdPublish (2016). 2018 May 9;7:97. [Version 1] doi: 10.15694/mep.2018.0000097.1 (PMC10712010; doi:10.15694/mep.2018.0000097.1)
Supplement: Peer review history

# Reviewer report: Reviewer response for version 1

**DOI:** 10.21956/mep.19574.r29044

**Published:** 2019-05-19

**Authors:** Ken Masters

**Affiliations:** 1 Sultan Qaboos University

This review has been migrated. The reviewer awarded 3 stars out of 5 An interesting paper investigating the factors affecting 1st-year medical students’ impressions of surgery as a career choice. The authors begin by outlining the problem of few medical students' choosing surgery as their specialty, and then assess their students’ impressions of surgery during a 2-week surgical exploration program. The background literature is well described, the results clearly presented, and the results are discussed in light of the literature. The authors do correctly identify some limitations as a rather small group of participants, and also that the study includes only students who already had an existing interest in pursuing a surgical specialty, so the generalisability of the results paper is limited.There are three other weaknesses of the paper:• The authors refer to the survey in Appendix A, but there does not appear to be an Appendix A.• Although the students’ demographic data (age and gender) are collected, these are not used (apart from a simple listing). The reasons for gathering this information would have been clearer if the authors had used it as part of their data analysis.• For such an explorative study, especially because of the small sample size, it would be been significantly strengthened with some qualitative investigation.So, an interesting and somewhat useful small study, within some limitations.

Reviewer Expertise:

NA

No decision status is available

# Reviewer report: Reviewer response for version 1

**DOI:** 10.21956/mep.19574.r29045

**Published:** 2018-05-09

**Authors:** Julie Hunt

**Affiliations:** 1 Lincoln Memorial University

This review has been migrated. The reviewer awarded 4 stars out of 5 Continuing to attract high-quality students to surgical disciplines is key. This article collects medical students' feedback about a surgery observation program to evaluate what in particular gives it educational value and personal enjoyment. The strengths of this study include the thoughtfulness and simplicity of its design and its interesting findings that contradict prior speculation about what has given value to similar programs.The authors' explanation for why positive regard and interactions proved significant could have been strengthened by the inclusion of educational theory. For example, self-determination theory suggests that optimal learning depends on autonomy, competence, and relatedness. Medical students had no autonomy in the cases. Some students had the opportunity to demonstrate competence while others did not; the value of competence is reflected in the nearly-significant value for student involvement in the case. All students had the opportunity for relatedness, which is the universal desire for connection, caring, and interaction, offering one explanation for why students rated team engagement and positivity so highly.

Reviewer Expertise:

NA

No decision status is available
